# The Clinical Accuracy of Diagnosing Chronic Conjunctival Lesions and the Importance of Limbal Involvement in Suspecting Malignancy

**DOI:** 10.3390/jcm15103784

**Published:** 2026-05-14

**Authors:** Ágnes Élő, László Tóthfalusi, Bianka Pencz, Alíz Pándi, Borbála Hornyák, Ágnes Füst, Olga Lukáts, Zoltán Z. Nagy, Nóra Szentmáry, Anna Énzsöly, Amarilla Barcsay-Veres

**Affiliations:** 1 Department of Ophthalmology, Semmelweis University, Maria Str. 39, H-1085 Budapest, Hungary; elo.agnes@semmelweis.hu (Á.É.); pandializ001115@gmail.com (A.P.); hornyak.borbala@stud.semmelweis.hu (B.H.); fust.agnes@semmelweis.hu (Á.F.); lukats.olga@semmelweis.hu (O.L.); nagy.zoltan.zsolt@semmelweis.hu (Z.Z.N.); szentmary.nora@semmelweis.hu (N.S.); bakos-kiss.anna@semmelweis.hu (A.É.); 2 Department of Pharmacodynamics, Semmelweis University, Nagyvárad tér 4, H-1089 Budapest, Hungary; tothfalusi.laszlo@semmelweis.hu; 3 Department of Pathology, Forensic and Insurance Medicine, Semmelweis University, Üllői út 93, H-1091 Budapest, Hungary; pencz.bianka@semmelweis.hu

**Keywords:** chronic conjunctival lesion, diagnostic accuracy, prevalence, histology, non-melanocytic conjunctival lesion, positive predictor value, limbal involvement

## Abstract

**Objectives**: Our objective was to evaluate the diagnostic accuracy of clinical diagnoses in patients with chronic conjunctival lesions (CCLs) in relation to prevalence data and to identify clinical features that improve diagnostic performance. **Methods**: Retrospective data were collected for all patients with CCLs at a tertiary eye clinic between 2006 and 2024. A total of 1304 patients were reviewed; 391 had available histopathological data. **Results**: Misclassification was the highest for clinically benign, non-melanocytic, non-degenerative CCLs (referral rates: 0.446; positive predictive value [PPV]: 0.289). The referral rates for histology assessment were comparable for malignant melanocytic and non-melanocytic CCLs (0.923 and 0.989, respectively) and for benign melanocytic and non-melanocytic CCLs (0.319 and 0.446, respectively). In contrast, the diagnostic agreement was double for melanocytic malignant CCLs (0.800 vs. 0.391) and triple for melanocytic benign CCLs (0.934 vs. 0.289) compared with non-melanocytic CCLs. However, the limbal involvement increased the odds ratio (OR) for proper diagnostic performance to 7.0 (95% confidence interval (CI): 2.7–19.1), whereas the limbal overlapping increased the OR to 11.2 (95% CI: 4.0–35.9) among malignant CCLs. **Conclusions**: The clinical diagnosis of non-melanocytic CCLs is less frequently and less accurately confirmed histologically. In this CCL subgroup, where the lesion involves or overlaps the limbus, the likelihood of malignancy is increased sevenfold to elevenfold.

## 1. Introduction

Chronic conjunctival lesions (CCLs) are persistent abnormalities of the conjunctiva that fail to resolve after more than 4 weeks. They may result from inflammatory, degenerative, infectious, or neoplastic processes. This thin mucous membrane, covering the anterior portion of the eye, may present with a diverse range of lesions [1,2,3,4,5,6]. There is a vast array of conjunctival disorders with clinical significance to identify beyond the rare pigmented conjunctival melanomas [6,7,8,9]. These lesions often present with prolonged conjunctival redness, thickening, pigmentation changes, or mass formation and require clinical evaluation to distinguish benign, premalignant, and malignant conditions. The potential symptoms include but are not limited to constant discomfort, inflammation, aesthetic problems, a perceived malignancy, and interference with contact-lens fitting [10,11]. Clinical recognition is mainly guided by slit-lamp examination and supported by a number of diagnostic imaging techniques [2,3,6,12,13], though a definitive diagnosis would come from histology and molecular assessments [8,14]. An early and accurate diagnosis is essential for proper management and patient information [11,15,16].

While the literature is abundant with papers describing the prevalence patterns of chronic conjunctival lesions [3,7,10,11,17,18,19,20], considerably less research has been conducted on how this clinical diagnosis is validated by histology and its implications for public health [13,21]. Furthermore, uncertainty remains over which clinical features should be considered to improve the accuracy of diagnosing individual conjunctival lesions [1,13,22]. Diagnostic evaluation of conjunctival lesions can be supported by impression cytology and histopathological biopsy or the non-contact imaging modality of anterior-segment OCT (Optical Coherence Tomography) [6,8,11,13]. The complex process of CCL diagnosis therefore involves mostly imaging modalities, and numerous machine learning models (a subset of artificial intelligence) are being developed to improve the accuracy and efficiency of image interpretation. However, the effectiveness of physicians in diagnosing CCLs has not been extensively researched. To create the most effective ‘intelligent assistant’ for future doctors diagnosing CCLs, it may be vital to highlight the areas of diagnostic performance that require the most assistance.

In addition to diagnostic advances, therapeutic strategies have also evolved. In selected cases—primarily in ocular surface squamous neoplasia (OSSN)—topical therapies such as interferon, mitomycin-C, or 5-fluorouracil may offer effective alternatives to complete surgical excision of conjunctival tumors [12,14]. Nevertheless, the clinical diagnosis remains pivotal, as it determines the subsequent diagnostic and therapeutic pathways. Therefore, the aims of our study were to evaluate the diagnostic accuracy of preliminary clinical diagnoses in patients with CCLs prior to histological assessment and to identify clinical features that influence diagnostic performance and a more judicious and evidence-based use of conjunctival surgery.

## 2. Materials and Methods

### 2.1. Study Design

A retrospective cohort study was conducted at the large Tertiary Eye Clinic at Semmelweis University (Hungary) between January 2006 and December 2024. Approval was obtained from the Regional and Institutional Committee of Science and Research Ethics at Semmelweis University (115-1/2023). This study was conducted in accordance with the Declaration of Helsinki.

### 2.2. Participants

The International Classification of Diseases (ICD-10) diagnosis codes were employed as a selection to delineate patient populations exhibiting chronic conjunctival lesions (2025 ICD10-CM Codes H00–H59: Diseases of the eye and adnexa, D31, C69, C.884).

The pre-screened medical records were analyzed individually. In the case of acute conjunctival lesions (e.g., lasting less than three weeks) or medical documentation that was inconsistent with the ICD-10 coding, exclusion was applied. Cases were not excluded on the basis of diagnostic uncertainty alone; all lesions with recorded clinical diagnoses—including those where the clinician expressed uncertainty—were retained in the dataset. This approach was chosen to reflect real-world clinical practice and to avoid introducing selection bias by removing diagnostically challenging cases.

Three clinicians diagnosed and operated on almost all cases. Further imaging techniques or laboratory tests may have been used if considered necessary in order to reach a definitive diagnosis. The characteristic features of the conjunctival lesions (largest basal diameter; presence of pigmentation; intrinsic tumor vascularity; feeder vessels; and cysts) were recorded. Limbal involvement was confirmed when a CCL originated from, infiltrated, or structurally destroyed the corneoscleral limbus (and associated stem-cell niche). Limbal overlapping was observed in cases where a conjunctival mass or redundant tissue folded or rested over the limbus and peripheral cornea without true cellular invasion, infiltration, or destruction. Slit-lamp photographs supported the data collection process.

Based on the WHO Classification of Tumors [23], the CCLs were combined into nine diagnostic categories, as follows, to compare the clinical and the histological diagnosis:

(1) Reactive and degenerative lesions (pterygium, pinguecula, scar, pseudopterygium, remnant of chronic bleeding, solar elastosis, lymphoid tissue, pyogenic granuloma);
(2) Non-melanocytic benign tumors (papilloma, apocrine hydrocystoma, haemangioma, syringocystoadenoma papillare, oncocytoma);
(3) Non-melanocytic malignant tumors (carcinoma planocellulare, sebaceous carcinoma, carcinoma squamocellulare Bowen-type, in situ carcinoma);
(4) Inflammations (granuloma, conjunctivitis lignosa, chronic conjunctivitis, rosacea, mixed inflammation);
(5) Lymphomas (mucosa-associated lymphoid tissue [MALT] lymphoma) and other non-Hodgkin-type low-grade lymphomas);
(6) Melanocytic benign tumors (subepithelial nevus, compound nevus, junctional nevus, blue nevus, Spitz nevus, benign epithelialis melanosis);
(7) Melanocytic malignant tumors (primary conjunctival melanoma, high-grade conjunctival melanocytic intraepithelial lesions, metastatic melanoma);
(8) Cysts (inclusion and implantation cyst, retention cyst, epidermoid cyst, infundibular cyst);
(9) Other lesions (orbital fat prolapse, lipodermoid, amyloidosis, lymphangiectasia).

One pathologist categorized all the histological findings into diagnostic groups. The histopathological assessment was conducted in accordance with standard methodologies.

### 2.3. Statistical Analysis

Descriptive statistics were calculated for all patients, including age, race/ethnicity, gender, and the CCLs, as well as the referral rate for histopathological examination. Sensitivity and specificity were not estimated, as histological verification was selectively performed based on clinical suspicion, rendering these measures non-informative without strong assumptions.

The prevalence of each CCL group was studied across age groups. The accuracy of the preliminary diagnoses compared to histopathological findings was expressed as the positive predictive value (PPV), defined as the proportion of patients with a positive clinical diagnosis confirmed by histology across diagnostic categories. To provide a measure of precision, 95% confidence intervals (CIs) for the PPV were calculated using the Wilson score method. PPV was selected as the primary metric because it directly addresses the clinical decision-making process in a referral setting (i.e., the probability that a suspected diagnosis is correct) and because the lack of histopathological verification for all lesions precludes the unbiased calculation of sensitivity and specificity. The association between PPV, histology requests, and clinical features that can enhance the PPV were also investigated. Univariate and multivariable logistic regression models were used to assess the association between confirmation of the clinician’s presumptive diagnosis (yes/no) and the presence of individual clinical features (yes/no). While univariate analyses were performed using available cases, missing data in the multivariable logistic regression models were handled using multiple imputation by chained equations (MICE) implemented with the mice package in R. The diagnostic accuracy in relation to the specific characteristics of conjunctival lesions was quantified using odds ratios (ORs) with the corresponding 95% confidence intervals. All analyses were performed using R software (R Core Team. Version 4.5.1. R Foundation for Statistical Computing, Vienna, Austria).

## 3. Results

Between January 2006 and December 2024, 1304 patient records were selected, relying on ICD10CM/coding. Following the validation of the medical records, 168 patients were excluded from further analysis due to incomplete data or ICD miscoding. The most common reason for exclusion was incorrect coding, meaning the clinical description did not match the selected diagnosis code. In approximately 2–3% of cases, the outpatient record was incomplete in terms of content, making further analysis impossible. The final cohort comprised 1136 patients (565 females, 571 males), with a mean age of 43.6 ± 23.1 years (range: 0.12–97.32 years), predominantly composed of Caucasian patients (99.13%), with minimal representation of patients from Arabic (0.43%), Asian (0.35%), and African (0.09%) ethnic groups.

### 3.1. Clinical Diagnostic Categories and Prevalence Data of the CCLs

**Figure 1:**
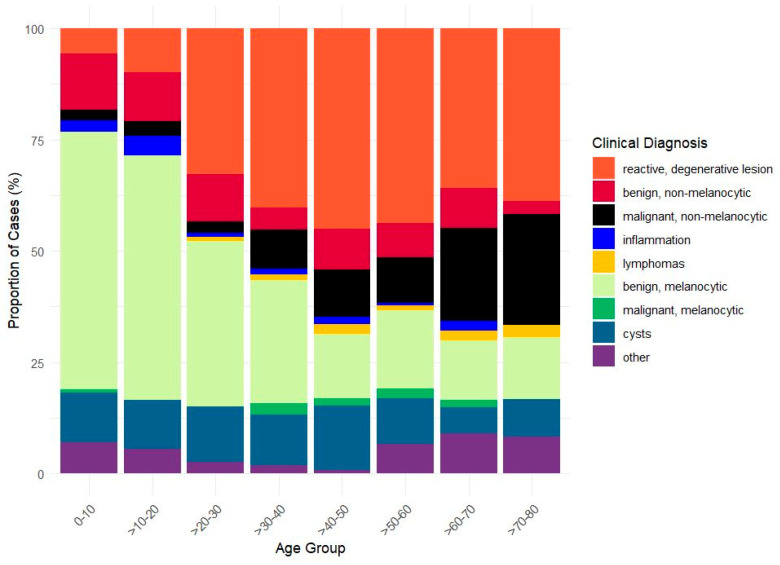
Clinical diagnostic categories by age group.

The most prevalent clinical diagnostic categories among the entire population were benign melanocytic lesions (33.63%), reactive degenerative lesions (28.44%), and cysts (10.57%), followed by benign non-melanocytic lesions (8.90%) and malignant non-melanocytic lesions (8.36%). Inflammation, malignant melanocytic cases, lymphomas, and other cases occurred with lower frequencies (1.58%, 1.14%, 1.05%, and 6.33%, respectively) (Figure 1, Table 1).

The benign melanocytic lesions predominate (0–10 yrs: 57.8%, 10–20 yrs: 54.9%, 20–30 yrs: 37.1%) during the first three decades of life. Thereafter, their prevalence gradually declines to 13.3% (>80 yrs group). Conversely, degenerative lesions show a progressive increase with age, reaching a prevalence of 40.2% between 30 and 40 years. Non-melanocytic malignant tumors can occur in patients of all age groups. Nonetheless, their proportion tends to increase with age, reaching its zenith in individuals over 80 years of age. Reactive, degenerative lesions dominated in patients over 40; notably, lymphomas and malignant melanocytic lesions manifested at a significantly lower frequency and predominantly occurred in individuals over 40 years of age. Moreover, in individuals over 70 years of age, the incidence of malignant melanocytic lesions surpassed that of benign melanocytic lesions. Cysts and benign non-melanocytic tumors showed similar proportions across all age groups (Figure 1).

### 3.2. Histopathology Referral Rate and Diagnostic Agreement

A total of 391 patients had histological diagnosis following a total (99.5%) or a subtotal (0.5%) excision procedure. The number of histology requests varied across the diagnostic groups. According to clinical judgement, the greatest need for histological examination was observed in malignant melanocytic lesions, malignant non-melanocytic lesions, and lymphomas (Table 1). The referral rate was defined as the number of histology requests divided by the total number of cases in each diagnostic category. The histological confirmation was not available in all cases, even when the presumptive diagnosis was a malignant tumor, as three patients declined surgery for personal reasons.

Detailed histopathological data are shown in Supplementary Table S1. In Table 1, a high agreement is shown between the preliminary clinical diagnosis and the gold-standard histopathological diagnosis among the lymphomas (PPV: 100%) and malignant melanocytic lesions (PPV: 80%). Similarly, other lesions (e.g., limbal dermoid, lymphangiectasia, etc.) or reactive, degenerative lesions (e.g., pinguecula, pseudopterygium, etc.) had high positive predictive values (>80%). In contrast, in the diagnostic group of non-melanocytic lesions, irrespective of whether benign or malignant, the positive predictive values were the lowest (approximately 30–40%) compared with all other diagnostic groups.

**Figure 2:**
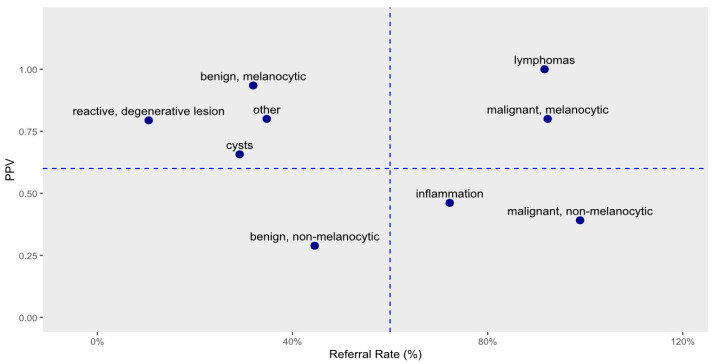
Diagnostic agreement vs. frequency of histology requests across clinical categories. Each point corresponds to a clinical diagnosis category. The x-axis represents the probability of requesting histology given a clinical diagnosis; the y-axis represents the PPV (diagnostic agreement) for that diagnosis. A dashed cross divides the space into four quadrants reflecting different diagnostic scenarios. n = 391.

Referral rates were comparable between malignant melanocytic and malignant non-melanocytic types (0.923 and 0.989, respectively), but the diagnostic agreement was more than twice as high for melanocytic lesions (0.800 vs. 0.391). Similarly, for benign lesions, referral rates were consistent across the melanocytic and non-melanocytic categories (0.319 and 0.446, respectively), yet the diagnostic agreement for melanocytic lesions was more than threefold higher (0.934 vs. 0.289). Figure 2 provides a graphical representation of the data shown in Table 1, illustrating the relationship between diagnostic agreement and referral rate for histology across clinical categories.

In Figure 2, the horizontal axis represents the referral rate for histology based on the clinical diagnosis, while the vertical axis shows the observed rate at which the diagnosis was confirmed. In Figure 2, a cross divides the plot into four quadrants, separating the clinical diagnostic categories accordingly. In the upper left quadrant, disorders are found for which histology is rarely requested; however, when histological data are available, the clinician’s diagnosis is correct with high certainty. The upper right quadrant contains malignant tumors. These are identified by clinicians with high confidence, but the seriousness of these diseases and current protocols necessitate histological confirmation. Ideally, 100% of these patients would have histological confirmation, but, as previously mentioned, three patients declined the recommended surgery. The lower right quadrant includes disorders where histological confirmation is frequently requested but the presumptive clinical diagnosis is confirmed less than 60% of the time. Finally, the lower left quadrant represents the most problematic group, which includes only benign non-melanocytic tumors. For this condition, the clinician’s diagnostic accuracy is just 28.9%, and histology is requested in only 31.9% of cases (see Table 1).

### 3.3. Special Features of Improving CCL Diagnostic Precision

**Figure 3:**
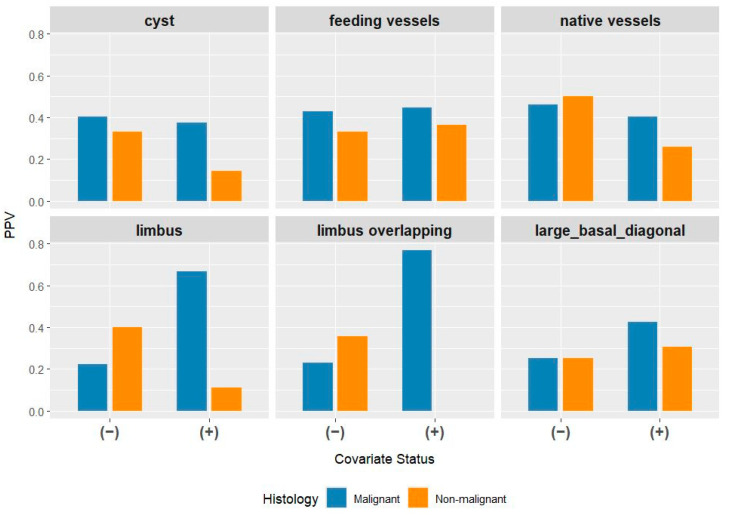
Clinical records for a subset of patients (n = 137) with non-melanocytic tumorlike lesions (benign or malignant) were reviewed for the presence or absence of additional clinical features, including limbal involvement (limbus), overlapping limbus, feeding vessels, native vessels, cysts, and a basal diameter larger than 2 mm. The left column duplet represents the absence; the right column duplet indicates that the particular feature is present.

Given the low diagnostic accuracy of the non-invasive screening method for non-melanocytic lesions, we investigated which clinical features and additional diagnostic criteria could assist clinicians in improving diagnostic precision. In this subgroup, in addition to slit-lamp examination, the clinical diagnosis was supported by anterior-segment OCT examination in 27 cases (19.7%). Among 137 patients with histologically confirmed non-melanocytic benign or malignant tumors, the presence or absence of the following characteristics was recorded by the clinician: limbal involvement (limbus), limbal overlapping (limbus overlapping), feeding vessels (feeding vessels), native vessels (native vessels), cysts, and a basal diameter greater than 2 mm (basal diameter > 2 mm). We calculated the PPVs stratified by the presence (+) or absence (–) of these features, separately for malignant and non-malignant tumors. Figure 3 presents the results, with six panels corresponding to the six diagnostic features. In each panel, the two left bars represent cases in which the diagnostic feature was absent and the two right bars represent cases in which the feature was present. Bar heights indicate the clinician’s diagnostic success rate (PPV), with colors denoting tumor type.

For most clinical features, no substantial differences can be observed when contrasting the malignant (left orange–blue bar duplets) with the non-malignant lesions (right orange–blue bar duplets), with the exceptions of limbal involvement and limbal overlapping in particular. The presence of limbal involvement or overlapping markedly increases the likelihood that the clinician’s presumptive diagnosis for malignancy will be confirmed by histology.

**Figure 4:**
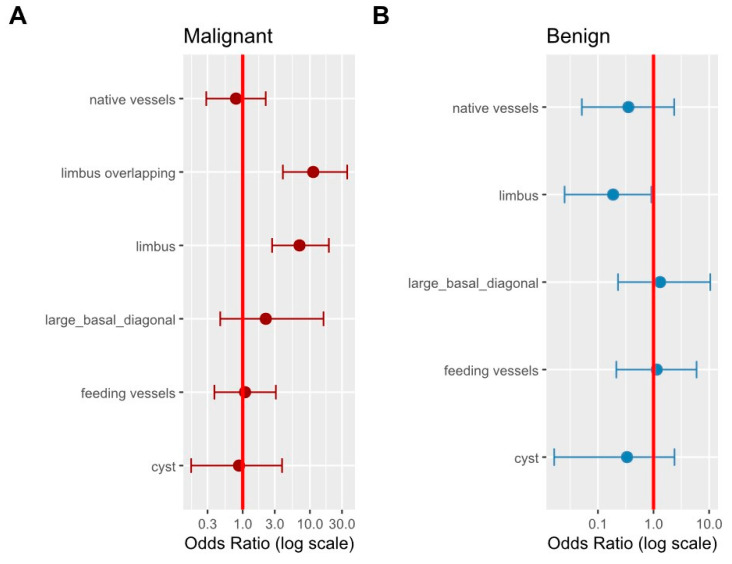
Univariate logistic regression analysis of anatomical and clinical features associated with the correct diagnosis of non-melanocytic conjunctival lesions. Odds ratios (ORs) and 95% confidence intervals (CIs) are shown. (**A**) Malignant lesions. (**B**) Benign lesions. In malignant lesions, limbal involvement and limbal overlapping were strongly associated with correct clinical identification, as their 95% CIs did not cross 1.

This visual impression is supported by univariate logistic regression analysis, which shows that if the non-melanocytic lesions reach or overlap the limbal area, the clinician’s odds of the correct identification of malignant non-melanocytic tumors increases severalfold (Figure 4A). Limbal involvement is a distinguishing characteristic of malignancy; malignant lesions involving the limbus were identified with an odds ratio (OR) of 7.0 (95% CI: 2.7–19.1), while overlapping the limbus increased the association further, with an OR of 11.2 (95% CI: 4.0–35.9). For non-malignant tumors, the PPV of the feature of limbal overlapping could not even be calculated due to the absence of relevant cases (Figure 4B). Conversely, in benign cases, the limbal involvement is a negative predictor: the PPV is significantly lower when a case is declared benign in the presence of limbal involvement compared to when it is absent. The upper limit of the 95% confidence interval does not cross the number 1 (Figure 4B).

In the non-pigmented non-melanocytic subgroup (n = 137), we performed multivariable logistic regression using histological malignancy as the outcome (Supplementary Table S3). After adjusting for patient age and clinical features (lesion size, vascularity, and cysts), only age reached statistical significance (OR = 1.04; 95% CI: 1.01–1.08; *p* = 0.021). While limbal involvement extending beyond the limbus showed directionally increased odds of malignancy (OR = 4.16), the resulting confidence intervals were very wide (95% CI: 0.81–21.42), indicating limited precision, likely due to the modest sample size relative to the number of parameters.

## 4. Discussion

Making a medical diagnosis is a complex decision, typically with gathering and integrating information. Medical diagnostic inaccuracies significantly impact quality of life, extend hospital stays, and increase morbidity and mortality, placing a significant burden on the healthcare system [24].

**Figure 5:**
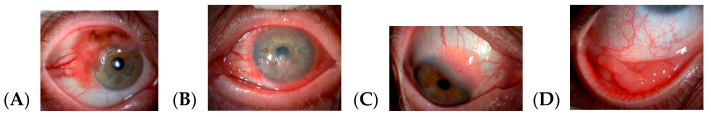
Slit-lamp photographs of CCLs: (**A**) malignant melanocytic melanoma, (**B**) planocellular carcinoma, (**C**) epithelial papilloma, and (**D**) MALT lymphoma.

This study explores the diagnostic considerations of chronic conjunctival lesions, encompassing clinical observations, imaging and histopathological analysis across a large cohort. Clinicopathologic correlation is a vital step in optimizing the outcomes and the appropriate treatment of a CCL [3,7,8] (Figure 5). The diagnosis of a CCL is the responsibility of numerous specialties (e.g., optometrist, eye specialist, general practitioner, or pediatrician). This study aimed to evaluate the accuracy of clinicians’ diagnoses in relation to CCL subtype prevalence data, identify areas for improvement in the current diagnostic process, and help determine the focus of future testing of AI systems.

Consistent with other previous studies [5,7,18,20,25], the benign melanocytic lesions posed the highest prevalence in the first four decades of life (exceeding 50%); later, the degenerative and the malignant lesions’ frequencies increased. The relative frequency of chronic conjunctival lesions varies according to age, as illustrated in Figure 1, based on 1134 conjunctival cases. This column diagram may serve as a particularly valuable resource for clinicians, guiding their diagnostic decisions. The highest absolute rate of histology requests was observed in the benign melanocytic lesion diagnostic group (Table 1), and the PPV of the preliminary diagnosis exceeded 93%. Regarding the concordance between the final histopathological diagnosis and the preliminary clinical diagnosis, Alkatan et al. [9] published a matching rate of 75.5% of 110 conjunctival specimens; the frequency of accurately matching diagnoses was higher among the benign lesions than the malignant entities (77% compared with 60%). Gruben et al. [21] analyzed 220 conjunctival tumors over a 9-year period, calculating the PPV based on the clinical description of the histological examination reports. The lowest PPV was identified for malignant (non-melanocytic) epithelial neoplasms at 0.65, which is similar to our results. However, a direct comparison is not feasible, as our diagnostic categories were more detailed (see Table 1). Cinotti et al. [13] compared the specificity of slit-lamp and in vivo confocal microscopy diagnosis in malignant conjunctival tumors (0.84 (95% CI: 0.63–0.95) vs. 0.77 (95% CI: 0.58–0.90)). In our study, malignant melanocytic tumors had the second highest PPV (0.80, see Table 1). According to our data, the clinical diagnosis of malignant melanocytic lesions was extremely rare before the fourth decade of age and the second most common diagnosis based on all histological analyses. Hence, histological examinations were performed in all cases, with the exceptions where the patients refused the procedure. From a clinician’s perspective, it is entirely justifiable to request the histological confirmation of a lesion suspected of being malignant, notwithstanding the potential futility of the procedure, as melanocytic lesions encompass nevus, racial melanosis, primary acquired melanosis, and melanoma, exhibiting a persistent challenge to diagnostic accuracy [1,6,7,8,25].

It is noteworthy that in contrast to the overall distribution of histological examinations, nonpigmented conjunctival lesions occurred at a comparatively lower frequency (benign: 11%; malignant: 24%), similarly to other studies [5,7,21]. Preliminary clinical diagnosis is followed by histological analysis in all cases of malignancy but in less than half of benign cases. However, irrespective of the histological classification of a lesion as benign or malignant, the positive predictive value of the clinician’s preliminary diagnosis is low. In our study, for the cases of non-pigmented, non-degenerative lesions, histological findings have been shown to confirm the original clinical diagnosis in approximately one in three cases (PPV: 0.391). Since the prevalence of non-melanocytic lesions remained stable at approximately 20% across all age groups, a considerable proportion of clinical diagnoses have limited predictive value and are often not validated upon referral for histology. Clinicians appear to be aware of these limitations, which may explain the negative trend between PPV and referral rate observed in Figure 2, except in the cases of malignant tumors.

We propose that the histology referral rate, reported alongside the PPV, constitutes a clinically meaningful parameter in its own right. Rather than being a mere methodological artifact of selective verification, the referral rate quantitatively reflects the clinician’s implicit risk–benefit assessment: a high referral rate signals perceived diagnostic uncertainty or clinical risk sufficient to justify surgical intervention, while a low referral rate indicates confidence in the clinical diagnosis or a judgement that the procedural risk outweighs the diagnostic benefit. Viewed in this light, Figure 2 demonstrates that clinicians are, on average, appropriately calibrated—referring more frequently in precisely those categories where their diagnostic accuracy is lowest. The notable exception—benign non-melanocytic lesions, which combine the lowest PPV (0.29) with a relatively low referral rate (44.6%)—may reflect underappreciation of diagnostic uncertainty in this subgroup and warrants particular attention in future clinical practice guidelines. The age difference observed between histologically verified and non-verified cases is most plausibly explained by the well-known association between advancing age and malignant lesion types rather than independent patient selection (Supplementary Table S2).

A substantial body of clinical studies provides evidence on the characteristics, frequency, and diagnostic difficulties of CCLs [1,3,26,27,28]. In a cohort of younger than 21 years [1], the relative risk of the comparative clinical features of benign vs. malignant conjunctival tumor counterparts were calculated. There was a higher risk for a malignant tumor regarding primary acquired melanosis (PAM) vs. malignant melanoma in cases of older age and with special morphologies, such as a larger size, presence of hemorrhage, intrinsic cysts, feeder vessels, and intrinsic vessels. Based on 262 patients’ data [1], a comparison between nevus and melanoma found that the relative risk for melanoma was increased almost fivefold in cases of tumor bases greater than 10 mm or lack of intrinsic cysts. In another cohort of 217 patients [28], malignant tumors displayed larger base diameters, intrinsic vessels and limbal locations. Based on our data of 391 histology assessments (see Figure 3 and Figure 4), the likelihood of malignancy is 7.0 times higher (95% CI: 2.7–19.1) when the limbus is affected and 11.2 times higher (95% CI: 4.0–35.9) when the tumor extends beyond the limbus among non-melanocytic, non-degenerative conjunctival tumors. The presence of limbal involvement or overlap is strongly associated with diagnostic accuracy in this subset of CCLs. Conversely, the absence of limbal involvement has been shown to reduce the odds ratio of malignancy by approximately 50%. The limbus is an anatomical site, approximately 1.5–2.0 mm wide, where the bulbar conjunctival epithelium transitions into the corneal epithelium [29]. This ensures that conjunctival cells do not proliferate into the corneal epithelium and mediates the physiological replacement of corneal epithelial cells and superficial wound healing via corneal epithelial stem cells [30]. The conjunctival-associated lymphoid tissue (CALT) plays a pivotal role in initiating and regulating ocular surface immune responses [31]. In cases of limbal involvement, not only the physical barrier but also the immune homeostasis may be compromised. This is not only the primary defense against microbes, but also, there is a direct association between inflammation and identifiable phases of immune dysfunction in the direction of tumorigenesis [32]. Melanocytic conjunctival melanoma’s location is 67% limbal as well [8,16]. Features such as feeding vessels, intrinsic vessels, cysts, and a basal diameter larger than 2 mm did not appear to be indicative of malignancy in our cohort with non-melanocytic, non-degenerative conjunctival lesions.

Previous studies have primarily focused on either the complete diagnostic task or, at most, the information-gathering subtask [33], leaving information-integration subtasks unexplored. We analyzed clinical records from a large ophthalmology clinic to characterize this decision-making process quantitatively, as limited data are available in the literature. The goal was to identify patients who may benefit from histological examination via conjunctival biopsy or excision for an accurate diagnosis. This could potentially shorten the time to diagnosis and allow for more judicious use of conjunctival surgery, thereby avoiding unnecessary surgery and expenses. Based on our data, identifying a malignant non-melanocytic tumor (noteworthily not a degenerative type of conjunctival lesion) poses a greater challenge in comparison with other chronic conjunctival lesions. The diagnosis of lymphomas demonstrated an exceptional degree of accuracy, and the non-melanocytic, non-degenerative CCLs exhibited the lowest. However, an early diagnosis facilitates the management of less severe cases, the employment of smaller diameter excisions, and fewer unnecessary operations or re-operations, resulting in enhanced clinical practice. In certain cases of chronic conjunctival lesions, wearing contact lenses is contraindicated [34].

This study has several limitations. First, our classification system focuses on clinical management rather than purely histological subtypes [1,7,23]. Second, the retrospective design, reliance on ICD-10 coding, and lack of histopathological verification for all lesions introduce potential verification bias. Further obscuring effects may have existed in exceptional circumstances when the clinical decision and the patient’s request were both contributing factors to the decision-making process regarding whether to proceed with excision or to arrange a subsequent follow-up. The near-homogeneous ethnic composition of our cohort (99.13% Caucasian) may be a limitation with respect to external validity. Histopathological verification was driven by clinical suspicion and lesion characteristics rather than applied randomly; consequently, sensitivity and specificity could not be reliably estimated without highly speculative assumptions regarding non-biopsied cases. Universal histological verification would be neither practical nor ethically justifiable in routine ophthalmic practice. The age difference observed between histologically verified and non-verified cases is most plausibly explained by the well-known association between advancing age and malignant lesion types rather than independent patient selection (Supplementary Table S2). Because PPV is prevalence-dependent, our results should be compared to other populations with caution. Finally, as a single-center study, our findings reflect the specific referral patterns of our institution.

Furthermore, the exclusion criteria applied in this study were based on objective criteria—acute lesion duration (less than three weeks) and ICD-10 miscoding—rather than on clinical ambiguity. Borderline or diagnostically uncertain cases were not formally excluded, which may have introduced a degree of heterogeneity within the diagnostic categories. This is inherent to retrospective chart review and reflects the reality of clinical practice, where diagnostic certainty varies. Future prospective studies should consider prespecified criteria for handling ambiguous cases to improve reproducibility.

Another limitation is the exploratory nature of the statistical analysis (Figure 4, Supplementary Table S3) within the non-pigmented non-melanocytic subgroup. Due to the modest sample size (n = 137), the multivariate logistic regression model included an unfavorable number of parameters relative to the available events, leading to limited statistical power and very wide confidence intervals for variables such as limbal overlap, precluding the identification of statistically significant independent associations. These findings highlight that while individual clinical signs show strong associations in univariate analysis, larger multicenter cohorts are required to reliably quantify their independent predictive value after adjusting for multiple clinical and demographic confounders.

## 5. Conclusions

In this retrospective cohort of 1136 chronic conjunctival lesion (CCL) cases, melanocytic lesions demonstrated substantially higher diagnostic agreement (PPV: 0.80–0.93) compared with non-melanocytic, non-degenerative lesions (PPV: 0.29–0.39), which represented the most diagnostically challenging category. Among non-melanocytic, non-degenerative CCLs, limbal involvement was associated with a 7.0-fold (95% CI: 2.7–19.1) and limbal overlapping with an 11.2-fold (95% CI: 4.0–35.9) increase in the odds of correct malignancy identification in univariate analysis. In this subgroup, the discrepancy between clinicians’ assumptions and histological findings was found to be the most significant. In order to achieve optimal clinical practice, it may be most beneficial for this subgroup to give due consideration to additional clinical features. These findings suggest that limbal involvement or overlap may serve as a clinically useful warning sign of malignancy in this subgroup.

However, given the retrospective design, the single-center setting, and the exploratory nature of the statistical analysis, these results should be regarded as hypothesis-generating. Prospective, multicenter validation is required before limbal involvement can be incorporated into formal management protocols.

## Figures and Tables

**Table 1:** Referral rates and PPVs by diagnostic category.

Diagnostic Category	Total Cases	Histology Cases	Referral Rate (%)	PPV (95% CI)
Reactive/degenerative lesion	323	34	10.5	0.79 [0.63–0.90]
Benign non-melanocytic	101	45	44.6	0.29 [0.18–0.43]
Malignant non-melanocytic	95	94	98.9	0.39 [0.30–0.49]
Inflammation	18	13	72.2	0.46 [0.23–0.71]
Lymphomas	12	11	91.7	1.00 [0.74–1.00]
Benign melanocytic	382	122	31.9	0.93 [0.88–0.97]
Malignant melanocytic	13	12	92.3	0.80 [0.52–0.94]
Cysts	120	35	29.2	0.66 [0.49–0.79]
Other	72	25	34.7	0.80 [0.61–0.91]

PPV = positive predictive value. Referral rate = histology-confirmed cases/total cases.

## Data Availability

The data presented in this study are available on request from the corresponding author due to privacy concerns.

## Supplementary Materials

The following supporting information can be downloaded at: https://www.mdpi.com/article/10.3390/jcm15103784/s1, Detailed histopathological data are shown in Supplementary Table S1. A baseline comparison between histologically verified and non-verified cases is provided in Supplementary Table S2. Exploratory multivariable logistic regression results for the non-pigmented non-melanocytic subgroup are shown in Supplementary Table S3.

## Abbreviations

The following abbreviations are used in this manuscript:

CLL	Chronic conjunctival lesion
MALT	Mucosa-associated lymphoid tissue
AI	Artificial intelligence
PPV	Positive predictive value
