# Supplementary material for: The Clinical Accuracy of Diagnosing Chronic Conjunctival Lesions and the Importance of Limbal Involvement in Suspecting Malignancy
Source: J Clin Med. 2026 May 14;15(10):3784. doi: 10.3390/jcm15103784 (PMC13207234; doi:10.3390/jcm15103784)
Supplement: Supplementary file 1 [file jcm-15-03784-s001.zip › S2_Table.pdf]

Table S2. Baseline characteristics by histopathological verification status

**Variable**	**Overall (N = 1143)**	**Histology (N = 390)**	**No histology (N = 753)**	**p-value**
Age (years)	43.6 ± 23.1 (median 45.6)	46.7 ± 23.7 (median 49.8)	42.0 ± 22.6 (median 42.4)	0.004
**Sex**				0.339
Male	572 (50%)	187 (48%)	385 (51%)	
Female	571 (50%)	203 (52%)	368 (49%)	
**Lesion type (clinical category)**				<0.001
Reactive, degenerative lesion	326 (29%)	35 (9%)	291 (39%)	
Benign tumor (non-melanocytic)	90 (8%)	45 (12%)	45 (6%)	
Malignant tumor (non-melanocytic)	107 (9%)	93 (24%)	14 (2%)	
Inflammation	19 (2%)	14 (4%)	5 (1%)	
Lymphomas	12 (1%)	11 (3%)	1 (0%)	
Benign melanocytic	382 (33%)	122 (31%)	260 (35%)	
Malignant melanocytic	13 (1%)	10 (3%)	3 (0%)	
Cysts	122 (11%)	35 (9%)	87 (12%)	
Other	72 (6%)	25 (6%)	47 (6%)	

p-values: Wilcoxon rank-sum test for age; chi-square test for sex and lesion type.
