# Supplementary material for: The Clinical Accuracy of Diagnosing Chronic Conjunctival Lesions and the Importance of Limbal Involvement in Suspecting Malignancy
Source: J Clin Med. 2026 May 14;15(10):3784. doi: 10.3390/jcm15103784 (PMC13207234; doi:10.3390/jcm15103784)
Supplement: Supplementary file 1 [file jcm-15-03784-s001.zip › SupplementaryTableHistology.pdf]

| **Histological categories in our cohort according to Shields et al.** | **n (%)** |
|---|---|
| **1. Inflammatory and Degenerative Lesions** | 50 (12.79) |
| Chronic conjunctivitis | 11 (2.81) |
| Phlyctenular keratoconjunctivitis | excluded |
| Pterygium | 16 (4.09) |
| Pinguecula | 14 (3.58) |
| Conjunctival scarring (e.g., post-inflammatory, cicatricial disease) | 9 (2.30) |
| **2. Reactive and Hyperplastic Lesions** | 16 (4.09) |
| Pyogenic granuloma | 12 (3.07) |
| Reactive lymphoid hyperplasia | 4 (1.02) |
| Chronic follicular conjunctivitis | 0 (0) |
| Giant papillary conjunctivitis | 0 (0) |
| **3. Cystic Lesions** | 30 (7.68) |
| Conjunctival inclusion cyst | 8 (2.05) |
| Retention cyst | 18 (4.60) |
| Parasitic cysts (rare) | 0 (0) |
| Other | 4 (1.02) |
| **4. Degenerative and Depositional Lesions** | 25 (6.39) |
| Conjunctival amyloidosis | 2 (0.51) |
| Elastotic degeneration | 5 (1.28) |
| Calcific degeneration | 0 (0) |
| Other | 18 (4.60) |
| **5. Benign Tumors with Chronic Presentation** | 188 (48.08) |
| Conjunctival nevus | 145 (37.09) |
| Squamous papilloma | 24 (6.14) |
| Hemangioma | 7 (1.79) |
| Dermoid / dermolipoma | 1 (0.26) |
| other | 11 (2.81) |
| **6. Premalignant and Malignant Lesions with Indolent Course** | 64 (16.37) |
| Primary acquired melanosis (PAM) | 6 (1.53) |
| Conjunctival intraepithelial neoplasia (CIN) | 13 (3.32) |
| malignant, epithelialis dysplasia | 4 (1.02) |
| Squamous cell carcinoma | 23 (5.89) |
| Conjunctival melanoma | 18 (4.60) |
| **7. Lymphoid and Systemic Disease–Related Lesions** | 18 (4.60) |
| Conjunctival lymphoma | 18 (4.60) |

| | |
|---|---|
| Systemic inflammatory disease–related conjunctival lesions eg. Sarcoidosis) | 0 (0) |
| Leukemic infiltration | 0 (0) |

**Supplementary Table S1.** Prevalences of the histological findings in our cohort (n=391)
Histological categories listed according to Shields et al.
