# Supplementary material for: The Clinical Accuracy of Diagnosing Chronic Conjunctival Lesions and the Importance of Limbal Involvement in Suspecting Malignancy
Source: J Clin Med. 2026 May 14;15(10):3784. doi: 10.3390/jcm15103784 (PMC13207234; doi:10.3390/jcm15103784)
Supplement: Supplementary file 1 [file jcm-15-03784-s001.zip › S3_Table.pdf]

Table S3 Multivariable logistic regression (imputed) — predictors of histological malignant non-melanocytic diagnosis

Clinical feature	OR	95% CI	p-value
Limbal involvement (yes/no)	1.67	0.33–8.60	0.534
Limbal overlap	4.16	0.81–21.42	0.087
Age (years)	1.04	1.00–1.08	0.038
Basal diameter > 2 mm	1.74	0.30–10.08	0.532
Feeding vessels	1.06	0.31–3.56	0.928
Intrinsic vascular network	0.65	0.17–2.47	0.521
Intrinsic cysts	0.55	0.10–3.15	0.498

Analysis set: all histologically verified cases in this sheet (N=137); outcome events (histological malignant non-melanocytic, hist_cat=3): 40. Missing predictors handled via multiple imputation (MICE, m=20); ORs pooled with Rubin’s rules.
